# Screen time, problematic screen use, and eating disorder symptoms among early adolescents: findings from the Adolescent Brain Cognitive Development (ABCD) Study

**DOI:** 10.1007/s40519-024-01685-1

**Published:** 2024-09-04

**Authors:** Jonathan Chu, Kyle T. Ganson, Alexander Testa, Abubakr A. A. Al-shoaibi, Dylan B. Jackson, Rachel F. Rodgers, Jinbo He, Fiona C. Baker, Jason M. Nagata

**Affiliations:** 1 Department of Pediatrics, University of California, San Francisco, 550 16th Street, 4th Floor, Box 0503, San Francisco, CA 94143 USA; 2 Factor-Inwentash Faculty of Social Work, University of Toronto, Toronto, ON Canada; 3 Department of Management, Policy and Community Health, University of Texas Health Science Center at Houston, Houston, TX USA; 4 Department of Population, Family, and Reproductive Health, Johns Hopkins Bloomberg School of Public Health, Johns Hopkins University, Baltimore, MD USA; 5 Department of Applied Psychology, Northeastern University, Boston, MA USA; 6 Department of Psychiatric Emergency and Acute Care, Lapeyronie Hospital, Montpellier, France; 7 School of Humanities and Social Science, The Chinese University of Hong Kong, Shenzhen, Guangdong China; 8 Center for Health Sciences, SRI International, Menlo Park, CA USA; 9 School of Physiology, University of the Witwatersrand, Braamfontein, Johannesburg South Africa

**Keywords:** Eating disorders, Adolescent health, Screen time, Problematic screen use

## Abstract

**Purpose:**

Emerging research evidence suggests positive relationships between higher screen time and eating disorders. However, few studies have examined the prospective associations between screen use and eating disorder symptoms in early adolescents and how problematic screen use may contribute to symptom development.

**Methods:**

We analyzed prospective cohort data from the Adolescent Brain Cognitive Development (ABCD) Study (*N* = 10,246, 2016–2020, ages 9–14). Logistic regression analyses were used to estimate the longitudinal associations between baseline self-reported screen time and eating disorder symptoms in year two. Logistic regression analyses were also used to estimate cross-sectional associations between problematic screen use in year two (either problematic social media or mobile phone use) and eating disorder symptoms in year two. Eating disorder symptoms based on the Kiddie Schedule for Affective Disorders and Schizophrenia (KSADS-5) included fear of weight gain, self-worth tied to weight, engaging in compensatory behaviors, binge eating, and distress with binge eating.

**Results:**

Each additional hour of total screen time and social media use was associated with higher odds of fear of weight gain, self-worth tied to weight, compensatory behaviors to prevent weight gain, binge eating, and distress with binge eating two years later (odds ratio [OR] 1.05–1.55). Both problematic social media and mobile phone use were associated with higher odds of all eating disorder symptoms (OR 1.26–1.82).

**Conclusions:**

Findings suggest greater total screen time, social media use, and problematic screen use are associated with more eating disorder symptoms in early adolescence. Clinicians should consider assessing for problem screen use and, when high, screen for disordered eating.

**Level of evidence:**

Level III: Evidence obtained from well-designed cohort or case–control analytic studies.

**Supplementary Information:**

The online version contains supplementary material available at 10.1007/s40519-024-01685-1.

## Introduction

Eating disorders are distressing and chronic disorders, linked to significant medical complications and reduced quality of life [1]. Examples of eating disorders include but are not limited to anorexia nervosa, bulimia nervosa, and binge-eating disorder, which are three of the most notable eating disorders among young people around the world [2]. The etiology of eating disorders is thought to be multifactorial. Studies have identified risk factors across biological, psychological, and sociocultural domains, such as genetic predisposition, elevated body mass index (BMI), comorbidity with other mental health disorders, socioeconomic status, and gender [3, 4]. Among these risk factors, a rising area of research is the relationship between screen use, the time spent using devices such as television, video game consoles, and mobile phones for various activities, and eating disorder risk [5, 6].

In recent years, the increasing popularity of social media has led to numerous studies describing the associations between social media use, body image, and eating concerns [7, 8]. However, the majority of these studies have occurred in mostly older female adolescents and young adults (approximately 15–29 years of age). While the focus on this age range is most likely related to the age of onset of clinical diagnoses, studies have shown that eating disorder symptoms may develop in early adolescence [9]. In addition, studies tend to focus on restrictive behaviors, excluding other symptoms of eating disorders such as compensatory behaviors (e.g., vomiting, excessive exercise) and binge eating and also eating disorder cognitions (e.g., feeling self-worth tied to weight, fear of weight gain, and distress with binge eating) that also make up DSM-5 criteria for eating disorder diagnoses [8, 10, 11]. The early adolescent population is known to also have increasing rates of screen use [12], and early symptom development may predispose individuals to long-term disordered eating [3, 13]. Furthermore, early adolescence is a key developmental period in which both the onset of puberty and increased social expectations impact mental health [14]. Therefore, it is imperative to further investigate these risk factors for eating disorders in younger populations to inform advancements in early identification and prevention.

Though the exact mechanisms through which screen time may influence the development of eating disorders is not yet fully understood, the Dual Pathways model describes how pressures to obtain socially constructed body ideals and subsequent body dissatisfaction increase the risk for negative eating disorder cognitions and disordered eating [15]. For example, increased exposure to idealized images of bodies on social media platforms (e.g., Facebook, Instagram, TikTok) may contribute to eating disorder symptoms in youth [16]. One cross-sectional study of 996 Australian adolescents with a mean age of 13 years linked increased social media usage with more disordered eating behaviors, suggesting that these influences may begin at younger ages [17]. However, the cross-sectional design of this study limited its generalizability regarding the directionality of the relationship and thus evidence for social media use as a risk factor for eating disorders. Therefore, longitudinal studies are needed to better understand media use as a potential risk factor for eating disorder symptoms in younger adolescents.

In addition to social media, it is also important to explore how other modalities of screen use, such as television, videos, video games, and texting factor into the potential development of eating disorder symptoms. One prior investigation found longitudinal associations between these screen time modalities and binge-eating disorder in early adolescents [6]. However, the eating outcomes assessed only included binge-eating disorder, and thus, individual symptoms characteristic of anorexia nervosa and bulimia nervosa, such as fearing weight gain, feeling self-worth tied to weight, and inappropriate compensatory behaviors/purging were overlooked. Increased screen time may influence emotional regulation in children and adolescents [5], and prior models have suggested strong associations between emotional dysregulation and eating disorders [18]. Thus, it is of importance to elucidate potential detrimental effects related to eating disorder risk.

Beyond the time spent using screens, further specific screen time behaviors and experiences should be investigated in relation to the risk of developing eating disorder symptoms. For example, problematic social media use, defined as the preoccupation with and compulsion to excessively engage in social media platforms [19], has been linked to deleterious outcomes in physical and mental health, including poorer mental health, sleep disturbances, and dietary problems [5]. Problematic mobile phone use shares similarities with problematic social media use and includes broader applications such as texting, apps, and video chatting. Studies have begun to examine the relationship between problematic screen use and negative eating habits and increased sedentary time [7, 20], suggesting that more problematic screen use is associated with higher body mass index [21, 22].

However, the associations between problematic screen use and eating disorder symptoms (e.g., body dissatisfaction) are less understood. In a large study of adolescents in Slovakia, eating disorder symptoms were associated with excessive internet use and potentially linked to poorer self-control and increased impulsivity [23]. As such, there may exist an overlap between the maladaptive behaviors and symptoms associated with eating disorders and the impulsivity related to problematic screen use. Additional cross-sectional studies have also shown similar relationships between problematic screen use and eating disorders symptoms, but have primarily been limited to smaller samples and older populations [10, 24, 25]. As the literature has shown that both problematic screen use and eating disorder symptoms may begin in early adolescence [9, 26], further studies are needed to potentially inform early prevention strategies.

The current study aimed to determine the prospective associations between total screen time and social media use at baseline and eating disorder symptoms (e.g., fear of obesity, feeling self-worth is tied to weight, engaging in compensatory behaviors, binge eating, distress with binge eating) at two-year follow-up in a large, national sample of early adolescents. Given the availability of problematic screen use data at the 2-year follow-up, the study also sought to determine the cross-sectional associations between problematic screen use (e.g., problematic social media or mobile phone use) and eating disorder symptoms. To better understand the specific relationship between these screen measures and eating disorder symptoms, we adjusted for potential confounders based on known risk factors, including sociodemographic factors (age, race/ethnicity, household income, parent education status), BMI, anxiety, and impulsivity [3, 26, 27]. We hypothesized that higher total screen time and social media use would be prospectively associated with reporting eating disorder symptoms [6, 16, 17]. We also hypothesized that problematic screen use would be cross-sectionally associated with eating disorder symptoms [24, 25].

## Methods

### Study population

We analyzed prospective data from the Adolescent Brain Cognitive Development (ABCD) Study, a longitudinal study of brain development and health across adolescence in 11,875 children recruited from 21 sites around the U.S. The ABCD study implemented epidemiologically informed strategies to recruit a sample representative of U.S. diversity, largely through school systems and considering sociodemographic factors. Additional details are described elsewhere [28]. Data analyzed are from the ABCD 4.0 release for the baseline (2016–2018, 9–10 years old), year one (2017–2019) and year two (2018–2020) assessments. Participants with missing data for screen time and eating disorder symptoms were excluded (*N* = 1,552, 13.1%, characteristics of included and excluded participants may be found in Additional file 1: Table S1). For participants missing sociodemographic data at baseline, including race/ethnicity, sex, household income, parental education, and study site, we implemented Gaussian normal regression imputation in Stata to impute missing data. Centralized institutional review board (IRB) approval was obtained from the University of California, San Diego. Study sites obtained approval from their respective IRBs. Caregivers provided written informed consent and each child provided written assent. Data used in this study were obtained from the ABCD Study (https://abcdstudy.org), held in the NIMH Data Archive (NDA).

### Exposures

#### Baseline total screen time and social media use

Total screen time and social media use were determined using the self-reported ABCD Youth Screen Time Survey. Participants answered questions about typical hours per day spent on six different screen time modalities (viewing/streaming television shows or movies, watching/streaming videos [e.g., YouTube], playing videogames, texting, video chatting [Skype, Facetime], and social media [e.g., Facebook, Instagram, Twitter]) separately for weekdays and weekend days, based on a previously validated measure [29, 30]. We calculated a weighted average of the participants’ typical weekday and weekend screen time use, ((weekday average × 5) + (weekend average × 2))/7, to report a single typical hours per day measure for each modality [22, 31]. We reported the weighted average as a continuous variable after obtaining this screen time total for each modality utilized by participants. Total screen time was determined by summing the weight averages of all modalities.

### Year-two problematic screen use

#### Problematic social media use

Starting in year two, the ABCD Study utilized the adolescent self-reported Social Media Addiction Questionnaire (SMAQ) to assess problematic social media. The six questions of the SMAQ were modeled after the Bergen Facebook Addiction Scale [32]. Examples of the questions included “I’ve tried to use my social media apps less but I can’t” and “I’ve become stressed or upset if I am not allowed to use my social media apps.” Likert-type scale responses ranged from 1 (never) to 6 (very often). Only participants who reported having at least one social media account were asked these items (*n* = 5,587).

#### Problematic mobile phone use

Starting in year two, a similar eight-question Mobile Phone Involvement Questionnaire (MPIQ) was used to assess problematic mobile phone use as reported by adolescents [33]. Examples of questions from the MPIQ included “I interrupt whatever else I am doing when I am contacted on my phone” and “I lose track of how much I am using my phone.” Likert-type scale responses ranged from 1 (strongly disagree) to 7 (strongly agree). This questionnaire has been previously used to assess smartphone dependence and digital multitasking during homework among US high school students [34]. Only participants who reported having mobile phones were asked these items (*n* = 7,280).

### Outcome: year-two eating disorder symptoms

The ABCD Study utilized the Kiddie Schedule for Affective Disorders and Schizophrenia (KSADS-5), a widely used computerized tool for categorizing child and adolescent mental health concerns based on the DSM-5, for assessment of eating disorder symptoms at two-year follow-up [35, 36]. Participants completed all modules of the KSADS-5 to assess the frequency, duration, and characteristics of eating disorder symptoms. Examples of questions participants were asked included “Do you feel like your self-worth is tied to your weight?” and “Was there ever a time, for a month or longer, that you worried all the time about your weight or becoming fat?” Participants were also asked about behaviors such as compensatory behaviors to lose weight and binge eating. Compensatory behaviors included only eating foods with minimal calories, exercising a lot, throwing up, and taking water pills, laxatives, or diet pills. Those who responded yes to any of the behaviors were coded as engaging in compensatory behaviors to lose weight. Participants were asked about binge eating and whether they experienced distress with binge eating. Additional information regarding the KSADS-5 assessment of eating disorder symptoms used in this study may be found in Additional file 1: Table S2.

### Confounders

We selected potential sociodemographic confounders based on previous literature and theory [3, 26, 27, 37]. Age (years), sex (female, male), race/ethnicity (White, Latino/Hispanic, Black, Asian, Native American, other), household income (grouped into two categories reflecting the US median household income: less than $75,000 and $75,000 or more), and highest parent education (high school or less vs. college or more) were based on parents’ self-report at baseline. Participant BMI was recorded at baseline. Measures of impulsivity were obtained using the Behavioral Inhibition and Approach Systems scale in the ABCD Study, which assesses participant reward responsiveness, drive, and fun-seeking behavior [38, 39]. Anxiety symptoms at baseline were obtained from parent/caregiver responses to the Child Behavior Checklist (CBCL), a screening tool used to assess psychiatric symptoms and behavior problems in children aged 4–18 [28, 40]. Because participants were asked about eating disorder symptoms at the year two assessment but not asked at baseline, we included parent-reported baseline eating disorder symptoms of their child based on the caregiver KSADS-5 assessment as a confounder in longitudinal analyses. ABCD Study site was included as a confounder to adjust for potential regional variation.

### Statistical analysis

Multiple logistic regression analyses were conducted using Stata 18.0 (StataCorp, College Station, TX) to (1) estimate prospective associations between screen time (exposure variable) and the presence of adolescent-reported eating disorder symptoms (fearing obesity, feeling self-worth tied to weight, engaging in compensatory behaviors to lose weight, and binge eating) at two-year follow-up, adjusting for confounders including parent-reported baseline eating disorder symptoms, and (2) estimate cross-sectional associations between problematic screen use and eating disorder symptoms, adjusting for confounders. Additionally, testing for interactions between eating disorder symptoms and sex was not statistically significant, and thus, we did not stratify by sex. Propensity weights developed by the ABCD Study were applied to yield estimates representative of the age, sex, and race/ethnicity distribution of US adolescents based on the American Community Survey from the US Census using the svyset and svy commands in Stata as described in the ABCD Study’s guide for population-based analysis [38].

## Results

Table 1 describes the sociodemographic characteristics of the 10,246 participants included. The sample was approximately matched by sex (48.6% female) and racially and ethnically diverse (45.6% non-White). At baseline, youth reported an average of 3.9 h of total screen time. At two-year follow-up, 1.4% reported fear of obesity, 1.6% felt their self-worth was tied to their weight, 0.7% engaged in compensatory behaviors to lose weight, 7.5% engaged in binge eating, and 2.9% had distress with binge eating.

**Table 1:** Sociodemographic, screen time, problematic screen use, and eating disorder symptoms among 10,246 Adolescent Brain Cognitive Development (ABCD) Study participants

Sociodemographic characteristics (baseline)	Mean (SD)/%
Age (years)	9.9 (0.6)
Sex, *n* (%)	
Female	48.6%
Male	51.4%
Race/ethnicity (%)	
White	54.4%
Latino/Hispanic	19.7%
Black	16.0%
Asian	5.4%
Native American	3.2%
Other	1.4%
Household income (%)	
Less than $75,000	45.0%
$75,000 and greater	55.0%
Parent with college education or more (%)	81.2%
Screen time measures	
Total screen time at baseline (hours)	3.9 (3.1)
Total screen time at year one of follow-up (hours)	4.7 (3.6)
Total screen time at year two of follow-up (hours)	6.1 (5.9)
Social media (hours)	0.1 (0.4)
Problematic screen use measures	
Social media addiction questionnaire score^a^	1.9 (0.9)
Mobile phone involvement questionnaire score^b^	3.1 (1.1)
Eating disorder symptoms	
Fear of obesity	1.4%
Self-worth tied to weight	1.6%
Inappropriate compensatory behaviors to prevent weight gain	0.7%
Binge eating	7.5%
Distress with binge eating	2.9%
BMI (kg/m^2^)	18.9 (4.2)
BMI percentile	61.6 (30.8)
Weight (kg)	28.0 (13.5)
Weight percentile	61.8 (29.7)
Anxiety symptoms (t-score)	53.7 (6.3)
BAS reward responsiveness sum score	2.2 (0.6)

Propensity weights were applied to yield representative estimates based on the American Community Survey from the US Census. SD = standard deviation

^a^Asked among a subset who reported social media use (*n* = 5,587)

^b^Asked among a subset who reported mobile phone use (*n* = 7,280)

Logistic regression analyses examining the prospective associations between baseline screen time and adolescent-reported eating disorder symptoms at two-year follow-up are shown in Table 2. In fully adjusted models, each additional hour of total screen time and social media use was prospectively associated with higher odds of fearing weight gain, feeling self-worth tied to weight, engaging in compensatory behaviors to prevent weight gain, binge eating, and distress with binge eating at two-year follow-up, with odds ratios ranging from 1.05 to 1.55.

**Table 2:** Associations between baseline total screen time and eating disorder symptoms at two-year follow-up in the Adolescent Brain Cognitive Development Study

Eating disorder symptom	Total screen time^a^		Social media use^a^	
	OR (95% CI)	*p*	OR (95% CI)	*p*
Fear of obesity	**1.12 (1.08–1.17)**	**< 0.001**	**1.55 (1.21–1.98)**	**0.001**
Self-worth tied to weight	**1.10 (1.06–1.15)**	**< 0.001**	**1.30 (1.03–1.63)**	**0.025**
Inappropriate compensatory behaviors to prevent weight gain	**1.06 (1.03–1.09)**	**< 0.001**	**1.18 (1.01–1.40)**	**0.039**
Binge eating	**1.08 (1.05–1.11)**	**< 0.001**	**1.28 (1.10–1.49)**	**0.002**
Distress with binge eating	**1.05 (1.01–1.09)**	**0.011**	**1.31 (1.06–1.61)**	**0.012**

Bold indicates *p* < 0.05

^a^Covariates: race/ethnicity, sex, household income, parent education, site, baseline parent-reported eating disorder symptom, baseline BMI percentile, baseline anxiety symptoms, and baseline BAS reward responsiveness

Table 3 shows logistic regression analyses examining the cross-sectional associations between problematic screen use (social media use or mobile phone use) and eating disorder symptoms at two-year follow-up. Both problematic social media use and problematic mobile phone use were associated with all eating disorder symptoms in fully adjusted models with odds ratios ranging from 1.26 to 1.82.

**Table 3:** Cross-sectional associations between problem screen time use and eating disorder symptoms in the Adolescent Brain Cognitive Development Study

Eating disorder symptom	Problematic social media use^a^		Problematic mobile phone use^a^	
	*n* = 5,587^b^		*n* = 7,280^b^	
	OR (95% CI)	*p*	OR (95% CI)	*p*
Fear of obesity	**1.38 (1.11–1.71)**	**0.004**	**1.43 (1.18–1.72)**	**< 0.001**
Self-worth tied to weight	**1.75 (1.45–2.10)**	**< 0.001**	**1.51 (1.27–1.79)**	**< 0.001**
Inappropriate compensatory behaviors to prevent weight gain	**1.43 (1.28–1.60)**	**< 0.001**	**1.26 (1.15–1.39)**	**< 0.001**
Binge eating	**1.63 (1.48–1.81)**	**< 0.001**	**1.66 (1.51–1.81)**	**< 0.001**
Distress with binge eating	**1.79 (1.54–2.08)**	**< 0.001**	**1.82 (1.57–2.12)**	**< 0.001**

Bold indicates *p* < 0.05

^a^Covariates: race/ethnicity, sex, household income, parent education, site, baseline BMI percentile, baseline anxiety symptoms, and baseline BAS reward responsiveness

^b^Assessments for problematic social media and mobile phone use were only performed on participants who responded "yes" to having a social media account or mobile phone, respectively

## Discussion

In this population-based, demographically diverse cohort of early adolescents in the US, we found that greater screen time and social media use were prospectively associated with eating disorder symptoms at two-year follow-up. We also revealed cross-sectional associations between problematic screen use and eating disorder symptoms. In particular, problematic social media use was most strongly associated with feeling self-worth tied to weight, and problematic mobile phone use was most associated with binge eating.

Our findings regarding the relationship between screen time, social media use, and eating disorder symptoms are consistent with prior studies [8, 10, 17]. While this relationship has been previously examined, longitudinal studies are scarce, particularly in younger adolescents, making this an important extension of previous work. Furthermore, as screen time and media use patterns rapidly evolve over time, continued studies are necessary to best capture their potential influence on youth growing up in different periods. Thus, we add to the literature by (1) using a large, national prospective cohort design; (2) focusing on early adolescence, an important period for the development of screen use and eating disorder symptoms; and (3) examining the associations between problematic screen use and eating disorder symptoms.

Of note, social media use only made up a small portion of total screen time in this population of early adolescents and had significant associations particularly with fear of weight gain. Through social media, youth may gain exposure to unrealistic beauty standards that could precipitate low self-esteem, leading to concerns regarding weight and body image [10, 17, 41]. The other forms of screen time that were not focused on in this study and which youth appear to be engaging with at higher amounts (e.g., television, videos, video games, texting) may also expose youth to similar content. Television shows and advertisements frequently depict and glamorize thinness in women and muscularity in men [42]. Influencers across various platforms, such as Instagram, YouTube, or TikTok have been shown to motivate and positively impact people’s exercise goals [43]; however, they often portray a “fit” ideal that may similarly lead to body dissatisfaction [20]. Future studies may seek to identify the relationships between specific screen time modalities and content that place youth at the greatest risk for developing eating disorder symptoms.

The relationship between problematic screen use and disordered eating is less well described in the literature, with existing studies primarily focusing on older adolescents, college students, and young adults [23, 25, 44]. In contrast to benign use, problematic screen use involves dependence and inability to remove oneself from screens, resulting in functional impairment in daily life. Prior studies have shown that problematic screen use and internet addiction may contribute to the development of poor eating habits [45]. For example, individuals may become so engrossed in their screen use that they unwittingly engage in disordered eating behaviors such as skipping meals to spend more time on their devices or binging due to a lack of awareness around how much they have eaten. Some preliminary studies have shown that mindful and intuitive eating practices, approaches to healthy eating that focus on non-judgmental observations of sensations and cognitions during meals, may reduce disordered eating behaviors [46]. As such, it may be possible that the decreased engagement during meals because of problematic screen use can predispose individuals to develop eating habits that then transform into disordered eating.

In our study, the association with the largest odds ratio was between problematic social media use and feeling self-worth tied to weight. Models describing the etiology of eating disorders often include environmental factors such as social pressure regarding physical appearance [47]. Social media has erupted in the last decade, resulting in increased connectedness to peers [48]; however, increased exposure may result in negative cognitions around body dissatisfaction, fearing obesity, and greater emphasis on body image due to social comparisons with content that embodies thinness ideals [10, 17]. Those who engage in problematic social media use are potentially more prone to constantly comparing themselves to other social media users at greater frequencies, which has been shown to have associations with body dissatisfaction and drive for thinness [16]. Consequently, it is possible that constant social media use can make adolescents more vulnerable to these body ideals and feelings of self-worth tied to their weight and body image.

In addition to these negative body image cognitions, we also found that problematic screen use was associated with binge eating and compensatory behaviors to prevent weight gain. Binge eating involves the overconsumption of food in a short period coupled with a loss of control during episodes. In a prior study, we showed that total screen time was longitudinally associated with binge-eating disorder. However, that study did not examine problematic screen use. Combined with purging, which are compensatory behaviors such as vomiting or excessive exercise to prevent weight gain, binge eating also contributes to bulimia nervosa as well as the binge–purge subtype of anorexia nervosa [49]. Theoretical frameworks attempting to explain the etiologies of these disorders have discussed the potential role of impulsivity [50, 51]. The seemingly impulsive nature of binge eating and purging may share similarities with characteristics of addiction and problematic screen use. Poor inhibitory control in impulsivity has well-established links to addictive behaviors [52]. Impulsivity generally refers to taking action or engaging in behaviors without consideration of consequences. High levels of impulsivity are thought to increase the risk of binge–purge episodes and have been demonstrated in longitudinal studies of adults as well as cross-sectional studies of adolescents [51, 53, 54]. Problematic usage and overconsumption of either social media or mobile phones may reflect the similar loss of control and overconsumption exhibited through binge-eating behaviors, which is consistent with our longitudinal findings between total screen time and eating disorder symptoms. Furthermore, children may be prone to overeating in the absence of hunger while distracted in front of screens. Finally, researchers posit that media and advertising content that youth may become exposed to can reflect unattainable body ideals and exacerbate binge eating [41], and adolescents who hold negative feelings towards their own body image are more likely to binge eat [55].

Our study includes notable limitations. Although we adjusted for several potential confounders, including parent-reported baseline eating disorder symptoms, the possibility of residual confounding due to other factors exists. Though the prospective study design for analyses between screen time and eating disorder symptoms improves on prior cross-sectional evidence, we cannot establish causality given the observational nature of the study. As the prevalence of eating disorder symptoms and the diagnoses of eating disorders increase as youth enter later adolescence, additional studies following the ABCD cohort will be an important area of future research. Furthermore, in this study eating disorder symptoms were assessed by parents at baseline and then adolescents at two-year follow-up since participants themselves were not screened for symptoms at baseline. Prior studies have demonstrated parents may provide lower estimates of eating disorder symptoms [56]; however, we acknowledge that generally, there exists discordance between youth–parent reporting of eating disorder pathology that future research may consider evaluating further [56]. Additionally, in this study eating disorder symptoms were analyzed categorically rather than dimensionally, which may not capture the relationship between screen use and the spectrum of symptom severity. It is important to note that the effect sizes of the associations between screen time and eating disorder symptoms were relatively small. However, they are reported for each additional hour, and thus, greater exposure may result in higher odds of developing symptoms. Despite the large sample size, participants in the study represent adolescents only within the US, which limits generalizability as both screen time and eating disorder patterns can vary in different regions globally [57, 58]. Because problematic screen use measures were not asked at the initial assessment of the ABCD Study, we were unable to determine the prospective associations between problematic screen use and eating disorder symptoms, though this may be another area of future research. Finally, all measures, including evaluations of screen time and eating disorder symptoms, were based on self-reported responses to survey questions and may be subject to reporting bias.

Given the ubiquitous nature of screen and media use in society and the mounting evidence for risks associated with their use, it is imperative to understand their potential downstream effects on youth. Especially with recent rises in both screen use and eating disorders [12, 59], future research should continue to examine their relationship in adolescent populations. Parent education regarding digital media literacy, which has been shown in some studies to decrease screen time in children, can potentially include guidance on body image concerns. The American Academy of Pediatrics encourages the development of Family Media Use Plans, which can include discussions surrounding problematic screen use and disordered eating concerns with children. Clinicians are encouraged to regularly assess screen time in youth, given the accumulating support for its association with a range of poor mental health outcomes. Moreover, clinicians should consider screening for disordered eating in youth who report high or problematic screen use, given the benefits of early identification for prognosis.

## Strength and limits

Strengths of the study include the analysis of a large, diverse prospective cohort of early adolescents in the US. Limitations include the use of self-reported measures which could be subject to reporting bias, the lack of problematic screen use measures at baseline, and the possibility of residual confounding.

## What is already known on the subject?

Emerging research evidence suggests positive relationships between higher screen time and eating disorders. However, few studies have examined the prospective associations between screen use and eating disorder symptoms in early adolescents and how problematic screen use may contribute to developing eating disorder symptoms.

## What does this study add?

Findings suggest greater total screen time, social media use, and problematic screen use are associated with more eating disorder symptoms in early adolescence. Clinicians should consider assessing for problem screen use and, when high, screen for disordered eating.

## Supplementary Information

Below is the link to the electronic supplementary material.

**Supplementary file 1.** **Table S1. **Comparison of characteristics between included and excluded participants, ABCD study. **Table S2. **KSADS-5 assessment of eating disorder symptoms in the ABCD study

## Data Availability

Data used in the preparation of this article were obtained from the ABCD Study (https://abcdstudy.org), held in the NIMH Data Archive (NDA).
