# Supplementary material for: Screen time, problematic screen use, and eating disorder symptoms among early adolescents: findings from the Adolescent Brain Cognitive Development (ABCD) Study
Source: Eat Weight Disord. 2024 Sep 4;29(1):57. doi: 10.1007/s40519-024-01685-1 (PMC11374868; doi:10.1007/s40519-024-01685-1)
Supplement: Supplementary file 1 — Table S1. Comparison of characteristics between included and excluded participants, ABCD study. Table S2. KSADS-5 assessment of eating disorder symptoms in the ABCD study [file 40519_2024_1685_MOESM1_ESM.docx]

| **Supplemental Table 1. Comparison of characteristics between included and excluded participants, ABCD study.** | | | |
| --- | --- | --- | --- |
| Sociodemographic characteristics | Included  (n= 10,246) | Excluded (n= 1,552) | p |
| **Age** | 9.9 (0.6) | 9.9 (0.6) | 93.3% |
| **Sex (%)** |  |  |  |
| Female | 47.7% | 49.0% | 34.4% |
| Male | 52.3% | 51.0% |  |
| **Race/ethnicity (%)** |  |  |  |
| White | 53.9% | 40.2% | <0.001 |
| Latino / Hispanic | 16.8% | 18.6% |  |
| Black | 18.6% | 30.2% |  |
| Asian | 5.9% | 6.4% |  |
| Native American | 3.5% | 3.0% |  |
| Other | 1.2% | 1.6% |  |
| **Household income (%)** |  |  |  |
| Less than $75,000 | 57.1% | 43.2% | <0.001 |
| $75,000 and greater | 42.9% | 56.8% |  |
| **Parent with college education or more (%)** | 84.4% | 72.2% | <0.001 |
| **Screen Time Measures** |  |  |  |
| Total Screen Time at baseline (hours) | 3.7 (3.0) | 4.329 (3.462) | <0.001 |
| Total Screen Time at year 1 of followup (hours) | 4.420 (3.462) | 4.952 (3.700) | <0.001 |
| Total Screen Time at year 2 of followup (hours) | 5.805 (5.643) | 4.893 (6.257) | 10.7% |
| Social Media (hours) | 0.108 (0.401) | 0.163 (0.536) | <0.001 |
| **Problematic Screen Use Measures** |  |  |  |
| Social Media Addiction Questionnaire Score^a^ | 1.837 (0.890) | 1.811 (0.857) | 84.6% |
| Mobile Phone Involvement Questionnaire Score^b^ | 3.072 (1.101) | 3.016 (1.146) | 70.5% |
| **BMI (kg/m**^2^) | 18.752 (4.143) | 19.375 (4.803) | <0.001 |
| **BMI percentile** | 60.351 (30.872) | 63.589 (30.637) | <0.001 |
| **Weight (Kg)** | 27.760 (13.439) | 29.071 (13.457) | <0.001 |
| **Weight percentile** | 61.212 (29.632) | 64.101 (29.672) | <0.001 |
| **Anxiety symptoms (t-score)** | 53.473 (6.090) | 53.573 (6.346) | 54.9% |
| **BAS reward responsiveness sum score** | 2.202 (0.580) | 2.190 (0.608) | 45.3% |

| **Supplemental Table 2. KSADS-5 Assessment of Eating Disorder Symptoms in the ABCD Study** | | |
| --- | --- | --- |
| **Symptom** | **Question** | **Comments** |
| Worry About Weight Gain | In the past two weeks, how often have you been preoccupied with your weight or worrying a lot about being fat? | Participants selected responses as follows: Not at all, rarely, several days, more than half the days, and nearly every day. Those who responded "nearly every day" were coded as worrying about weight gain. |
| Self-Worth Tied to Weight | Do you feel like your self-worth is tied to your weight? | Participants who responded "yes" were coded as feeling self-worth tied to weight. |
| Inappropriate Compensatory Behaviors to Prevent Weight Gain | Please note below all the different methods that you have used to control your weight. | Participants were asked to select from the following behaviors: diet pills, laxatives, water pills, throwing up, exercising a lot, only eating foods or drinks with minimal calories (e.g., carrots, celery, zero calorie drinks), or other (fill in). Those who selected at least one response option were coded as engaging in inappropriate behaviors to prevent weight gain. |
| Binge Eating | In the past two weeks, how often have you had eating binges, when you lost control of your eating and ate way more than you needed, because you were unable to stop yourself from eating? | Participants who responded "yes" were coded as binge eating. |
| Distress With Binge Eating | How much discomfort or distress does binge eating cause you? | Participants were asked to select a response ranging from 0 to 10. The Kiddie Schedule for Affective Disorders and Schizophrenia (KSADS-5) assessment used a response of 3 or higher as the cutoff to indicate having distress with binge eating. |
